# The viable but non-culturable (VBNC) status of *Shewanella putrefaciens* (*S. putrefaciens*) with thermosonication (TS) treatment

**DOI:** 10.1016/j.ultsonch.2024.107008

**Published:** 2024-07-30

**Authors:** Ziwei Jiang, Yi Wang, Shunjie Bai, Chan Bai, Ziyi Tu, Hailan Li, Peng Guo, Tao Liao, Liang Qiu

**Affiliations:** a Key Laboratory of Cold Chain Logistics Technology for Agro-Product, Ministry of Agriculture and Rural Affairs/Institute of Agro-Product Processing and Nuclear Agricultural Technology, Hubei Academy of Agricultural Sciences, Wuhan 430064, China; b Hubei Engineering Research Center for Agro-Product Irradiation, Agro-product Processing Research Sub-center of Hubei Innovation Center of Agriculture Science and Technology, Wuhan 430064, China; c School of Chemistry and Environmental Engineering, Wuhan Institute of Technology, Wuhan 4300731, China; d HuBei Crawfish Industrial Tech Ltd., Qianjiang 433100, China

**Keywords:** *Shewanella putrefaciens*, Thermosonication treatment, VBNC status, Protein regulation

## Abstract

Although thermosonication (TS) treatment has been widely used in food sterilization, the viable but non‐culturable (VBNC) of bacteria with TS treatment has still concerned potential food safety and public health. The molecular mechanism of VBNC status of bacteria with TS treatment is not clearly known. Therefore, in this study, we used *Shewanella putrefaciens*, which was a common putrefactive bacteria in aquatic products, to study the VBNC state of bacteria with TS treatment*.* Firstly, our results revealed that *S. putrefaciens* still could enter the VBNC state after TS treatments: 50 kHz, 300 W, 30 min ultrasonic treatment and 70 °C heating; Subsequently, we found the VBNC state of *S. putrefaciens* can resist the damage of TS treatment, such as cell wall break, DNA degradation, etc; Finally, four-dimensional data-independent acquisition-based proteomics showed that under VBNC state, *S. putrefaciens* upregulated functional proteins to resist TS treatment, such as: ribosomal proteins to accelerate the synthesis of stress proteins to counteract TS treatments, ornithine decarboxylase SpeF and MraY to repair TS treatment-induced damage, etc. Meanwhile, *S. putrefaciens* downregulates metabolic and transport functional proteins such as dehydrogenase to reduce the metabolism. Importantly, among those proteins, the ribosomal transcriptional regulatory protein family, such as rpsB, etc, may be the key proteins for *S. putrefaciens* entering VBNC state. This finding can provide some new strategies for preventing VBNC status of bacteria with TS treatment, such as: inhibition of key proteins, etc.

## Graphical abstract

**Figure:**
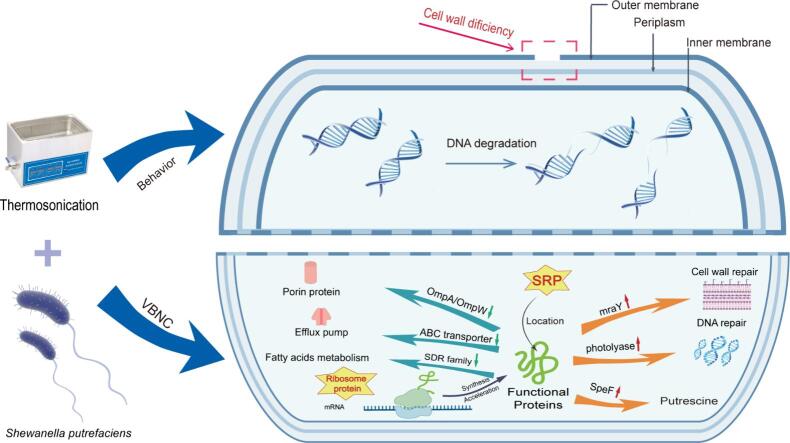

## 1 Introduction

Food safety poses a significant challenge to human survival; controlling microbial contamination is an effective measure to reduce food safety risks [1], [2]. *Shewanella putrefaciens* is a specific spoilage bacteria in most aquatic products, this gram-negative bacterium is highly tolerant to environmental changes and has a high capacity for putrefaction and pathogenicity [3]. Many sterilization methods including: heating; radiation; ultrasonic etc, have been studied for their bactericidal capacity for *S. putrefaciens*, aiming to guide future practical applications [4], [5], [6]. Thermosonication (TS) treatment is a combination of ultrasonic and heating method, reports have been studied the application of ultrasound in different foods and culture media under controllable temperatures, and it has been proven to be effective against foodborne pathogens and putrefactive flora. Owing to its controllability without changing the quality of products, TS treatments is considered to be an excellent substitute for heat treatment [7], [8].

However, due to the self-adaptive mechanism, during sterilization processes (including TS treatment), some microorganisms opt to adopt a life form characterized by low metabolic activity yet high resistance to ensure their survival. Such forms include spores, viable but non-culturable (VBNC) cells, and persisters [9], [10]. Among these, the VBNC state represents a dormant status adopted by non-spore-forming bacteria when facing adverse environmental conditions. Presently, over 100 microbial species, including various foodborne pathogens and spoilage bacteria, have been found capable of entering the VBNC state [11], [12]. Cheng et al. found that citral and *trans*-cinnamaldehyde induced *Staphylococcus aureus* (*S. aureus*) to enter the VBNC state, although the bacteria could still recover in tryptic soy broth (TSB) [13]. Zhang et al. found that even with prolonged exposure, some *S. aureus, Salmonella* and *Escherichia coli* strains enter the VBNC state when exposed to TS treatment [14]. Researches have shown that traditional bacterial culture methods are unable to detect the VBNC state, such as plate spreading [15]. This leads to a significant underestimation of bacterial content in food, and bacteria in the VBNC state still pose risks of food spoilage and pathogenesis [16].

The main characteristics of VBNC state microorganisms include: changes in cell morphology, reduced metabolic activity, increased resistance to external stimuli, and alterations in protein expression [17]. Zhao et al. used proteomics to study *Escherichia coli* (*E. coli*) induced into the VBNC state by high-pressure CO_2_, inferring that VBNC cells repaired membrane integrity and DNA damage while showing overall reduced metabolic activity and decreased pathogenicity [18]. Heim et al. compared the VBNC state of *Enterococcus faecalis* under starvation conditions using two-dimensional gel electrophoresis, revealing that although the overall metabolic level of VBNC cells was downregulated, proteins related to protein synthesis were upregulated, and the expression of proteins related to energy metabolism was altered, indicating that the VBNC state is a protective mechanism for bacteria in adverse environments [19]. Proteomic analysis of *Vibrio parahaemolyticus* in the VBNC state revealed upregulation of mainly non-metabolism-related genes and proteins involved in multiple pathways, such as transcription, translation, ATP synthesis, gluconeogenesis, and antioxidation [16].

Although researchers have demonstrated that bacteria can enter the VBNC state under TS treatment [20], the specific mechanisms at the protein level still remain unclear. Proteins are the foundation of biological matter and are directly associated with vital life phenomena [21]. Identifying essential proteins that regulate bacterial entry into the VBNC state is pivotal for targeted control strategies.

Therefore, this study used various biological methods, such as proteomics and flow cytometry, to explore how *S. putrefaciens* regulates itself to entry into the VBNC state under TS treatment. First, flow cytometry combined with trypan blue staining was used to verify the presence of *S. putrefaciens* in the VBNC state with TS treatment; then, using transmission electron microscopy (TEM), etc, to analyze the performance of *S. putrefaciens* after TS treatment; Finally, four-dimensional data-independent acquisition (4D-DIA)-based proteomics was employed to study the differential proteins of *S. putrefaciens* after TS treatment. This study aims to elucidate the potential molecular mechanisms of *S. putrefaciens* entering the VBNC state after TS treatment and to provide new insights into targeted inhibition of VBNC state bacteria in TS treatment applications.

## 2 Materials and methods

### 2.1 Materials

Freeze-dried *S. putrefaciens* strain CICC 22940 was purchased from the China Center of Industrial Culture Collection (Qingdao, China), and inoculated into Tryptic Soy Broth (TSB) (Qingdao Hope Bio-Technology, China). After twice activation by the plate scribe method, the colonies were picked and incubated at 30 °C for 8 h to reach the logarithmic phase. The bacterial cells were then collected by centrifugation and washed twice with sterile water. Following resuspension in phosphate-buffered saline (PBS, pH=7.2), the absorbance was adjusted to 600 nm to achieve a bacterial solution concentration of 10^8^ CFU/mL. This suspension was used to prepare samples for subsequent experiments. PBS solution was prepared using anhydrous potassium dihydrogen phosphate (0.21 g/L), anhydrous potassium hydrogen phosphate (0.325 g/L), and sodium chloride (9.0 g/L), with a final pH of 7.2. The remaining reagents are of analytical purity.

### 2.2 Sterilization of the *S. putrefaciens* by TS treatments

The bacterial suspension was exposed to ultrasonic device (KQ-800KDB, Kunshan Ultrasonic Instrument Co., ltd., Jiangsu, China) for 30 min under experimental conditions that included eight different temperatures (35℃, 40℃, 45℃, 50℃, 55℃, 60℃, 65℃, 70℃) and five different power levels (100 W, 150 W, 200 W, 250 W, 300 W). Each experimental group was named using the format: TS-Temperature-Power, resulting in a total of 40 groups (TS-35–100 to TS-70–300). Finally, the bacterial suspension was serially diluted in a 10-fold gradient using sterile PBS solution, we then removed 100 μL of appropriately diluted bacterial suspension from all groups for subculture on tryptic soy agar (TSA) plates (Qingdao Hope Bio-Technology Co., Ltd., China) at 30 °C for 24 h.

### 2.3 Staining and flow cytometry analysis

After different TS treatments, bacterial suspensions were followed by centrifugation (5810R, Eppendorf). Then, the bacterial cells were washed with 0.85 % sterile saline, resuspended, incubated with 10 μL SYTO 9 Green Fluorescent Nucleic Acid Stain (S34854, Invitrogen) and 60 μL propidium iodide (PI) (C0080, Solarbio) at room temperature in the dark for 15 min separately, and immediately analyzed using the flow cytometer (Guava EasyCyte, Luminex).

0.04 % trypan blue staining (T8070, Solarbio) solution was prepared and used to stain *S. putrefaciens* treated after different TS treatments for 3 min. Subsequently, the stained bacteria were resuspended in PBS to wash off the staining solution and evenly dispersed. After slide preparation, bright-field microscopy was performed to assess the survival status of the treated bacteria.

### 2.4 Resuscitation assay of VBNC strain

According to Hu’s and Yang’s methods [22], [23], the bacteria were treated with TS (TS-70–300), the VBNC state of the cells was confirmed by performing pour-plate assays on TSA plate, which showed 0 CFU/mL for culturable cell counts, then washing the bacteria with sterile PBS, resuspend it in TSB medium and blow it evenly. They were cultured in the shaker at 30 °C, and the absorbance at 600 nm was measured every 2 h; three independent experiments were performed.

### 2.5 Transmission electron microscope (TEM) analysis

After different TS treatments, the bacterial cells were collected by centrifugation and control groups (without treatment) were also prepared. Bacterial cells placed in 2 mL microcentrifuge tubes, mixed well with 2 mL of 2.5 % glutaraldehyde fixative solution, and fixed in a refrigerator at 4 °C for 12 h. The bacterial cells were then fixed with a 1 % osmic acid solution for 1 h, the osmic acid waste was carefully removed, and the samples were rinsed three times with PBS for 15 min each time. Next, the samples were dehydrated sequentially in 30 %–50 %–70 %–80 %–95 % acetone for 10 min each, followed by two 20-min rounds of dehydration in 100 % acetone. An embedding resin was poured into embedding molds, and the samples were inserted into the molds and baked at 60 °C for 48 h in an oven. The resulting polymerized resin blocks were removed from the molds and sliced into ultrathin sections (70–90 nm) using an ultramicrotome. The sections were picked with copper grids and stained with uranyl acetate for 10 min and lead citrate staining for 8 min. Finally, the samples were observed using a transmission electron microscope (Hitachi-7800, Japan), and images were captured for analysis.

### 2.6 Amplification of the internal transcribed spacer region sequence *S. putrefaciens*

*S. putrefaciens* DNA (1 μL) was exposed under different TS treatments. Then, the volume was adjusted to 50 μL using ultrapure water, thoroughly mixed, and allowed to react at room temperature for 20 min. Using this as the DNA template, the internal transcribed spacer region of *S. putrefaciens* was amplified by real-time quantitative polymerase chain reaction (qRT-PCR) using specific sense (5ʹ-TTCTTTGCCAATAAAGACAGATCTC-3ʹ) and antisense (5ʹ-GCAAGCTCTGGTTAGTTAATTCTTT-3ʹ) primers [24]. The PCR cycling conditions were as follows: initial denaturation at 94 °C for 2 min; 35 cycles of denaturation at 94 °C for 30 s, primer annealing at 55 °C for 30 s, and elongation at 72 °C for 30 s; and final extension at 72 °C for 2 min. The PCR products were visualized following 1 % agarose gel electrophoresis with GoldView I nucleic acid dye (G8140, Beijing Solarbio Science & Technology, China), observed under UV light and photographed.

### 2.7 4D-DIA proteomics

#### 2.7.1 Protein extraction, quantification, and enzyme digestion

Total proteins of *S. putrefaciens* in the VBNC state were extracted using B-PER Complete Bacterial Protein Extraction Reagent (89821, Thermo Fisher Scientific, USA) to lyse the cells. Following protein isolation, the total protein concentration was determined using the bicinchoninic acid method. The samples were then aliquoted and stored at −80 °C [25].

Protein samples (100 μg) were supplemented with 60 μL lysis buffer, followed by the sequential addition of triethylammonium bicarbonate to achieve a final concentration of 100 mM. Then the mixture was reduced with Tris (2-carboxyethyl) phosphine to a final concentration of 10 mM, and allowed to react at 37 °C for 60 min. After adding 40 mM iodoacetamide, the reaction mixture was kept in the dark at room temperature for 40 min. Next, precooled acetone (acetone:sample [v:v] = 6:1) was added to each reaction and incubated at −20 °C for 4 h. After centrifugation at 10,000×*g* for 20 min, the precipitate was collected and fully dissolved in 100 μL 100 mM tetraethylammonium bromide. Trypsin (Enzyme activity ≥250 units/mg) was added at a mass ratio of 1:50 (enzyme:protein) for overnight digestion at 37 °C. The peptide fragments were dried using a vacuum pump and then redissolved in 0.1 % trifluoroacetic acid, deslated using hydrophilic–lipophilic balance cartridges, and dried using a vacuum concentrator. Quantification of the peptide segments was performed using a peptide quantification kit (23275, Thermo Fisher Scientific, USA).

#### 2.7.2 Spectrum library establishment by data-dependent acquisition (DDA)

The digested peptides that were concentrated by vacuum centrifugation were redissolved in UPLC sample buffer for UPLC separation using a reverse-phase C_18_ column. Twenty fractions were collected based on peak shape and time. After vacuum centrifugation, the fractions were dissolved in mass spectrometry sample buffer and analyzed by mass spectrometry using DDA mode.

Proteome Discoverer 2.0 software (Thermo Fisher Scientific, USA) was used for database searching by submitting raw files to the Proteome Discoverer server after selecting “*Shewanella putrefacien*s”.

#### 2.7.3 DIA mass spectrometry detection of samples

Equal amounts of peptide segments were dissolved in mass spectrometry sample buffer (2 % ACN with 0.1 % formic acid). After adding 10 × iRT peptide segments in proportion and mixing evenly, the DIA detection analysis was performed, with DIA mode selected for data acquisition.

Proteome Discoverer software was used for database search and analysis of the fractionated library data. The search results were imported into Spectronaut software (Biognosys, USA) to establish the spectrum library. Spectronaut software was used to extract peptide peaks from DIA raw data based on ion-pair information from the spectrum library. The analysis used default software settings, iRT calibration, the selection of six specific peptide segments for each protein, selection of six fragment ions for each segment, and quantification based on their summed areas. Finally, a list of proteins present in the sample was obtained, including qualitative and quantitative data.

#### 2.7.4 Bioinformatics analysis

Gene ontology (GO; http://geneontology.org/) was used for the functional annotation of all differential proteins, and the Kyoto Encyclopedia of Gene and Genomes (KEGG) pathway database (http://www.genome.jp/kegg//) was used to analyze the metabolic pathways associated with the differential proteins.

The Fisher exact test in Goatools software was used to perform GO enrichment analysis of the protein set to determine their main GO functions. Adjusted P values (p-adjust) <0.05 were considered indicative of a significantly enriched GO function. KEGG pathway enrichment analysis of the protein set was performed using Majorbio Cloud platform (https://cloud.majorbio.com). The calculation principle was the same as that for the GO functional enrichment analysis, with p-adjust values <0.05 considered indicative of significant enrichment of the KEGG pathway function. The protein–protein interaction (PPI) network was constructed using the network modeling method. The topological attributes of the network were analyzed according to the network level, and the interaction relationship between essential proteins was revealed.

### 2.8 Statistical analysis

All experiments were conducted in three parallel groups. Data were expressed as mean ± SD, and analysis of variance was performed using the Duncan multiple range test in SPSS v20.0 statistical analysis software (IBM Corp., USA). P values <0.05 were considered statistically significant.

## 3 Results and discussion

### 3.1 Sterilization of the *S. putrefaciens* by TS treatments

In our previous studies and other results, time is not an important factor in TS treatments [26], [27]. In general, after 30 min, the bacteria had been treated [14]. Thus, in this experiment, we investigated parameters, including power and temperature as stress factors for studying the induction of VBNC state in *S. putrefaciens*.

**Fig. 1:**
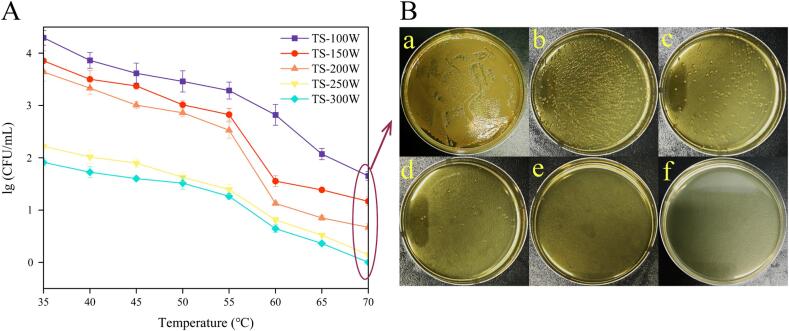
Colony counting results (A) and TSA plate culture results (B) of *S. putrefaciens* after TS treatment with different ultrasonic power and temperature*.* TSA plate culture results were the control group for normal growth without TS treatment (a), ultrasonic power of 100 W (b), 150 W (c), 200 W (d), 250 W (e), and 300 W (f) at 70℃.

In general, bacterial survival counts were reduced simply by increasing the power, accelerating the temperature, and prolonging the treatment time. As shown in Fig. 1, when *S. putrefaciens* was exposed to temperatures below 60 °C, the bacteria exhibited relatively high survival rates regardless of power changes, likely due to predominant ultrasonic sterilization at temperatures below 60 degrees. As the temperature increased, heat sterilization gradually became more effective.

However, as the conditions became more stringent, the adjustment mechanisms of the bacteria reached its limit, resulting in rapid bacterial death. Notably, when the power reached 250 W and the temperature was 70 °C, the number of *S. putrefaciens* had already decreased below the detection limit (≤1.4 lg (CFU/mL)). When the power reached 300 W, there was no observable colony growth on the plate. (Fig. 1B). Additionally, results from other studies showed that *Salmonella Typhimurium* required 380 W power and 53 °C to reduce the number of free bacteria below the detection limit [20]. This likely due to the thicker cell wall of gram-positive bacteria, which confers stronger resistance to TS treatment [28].

### 3.2 VBNC state of *S. putrefaciens*

**Fig. 2:**
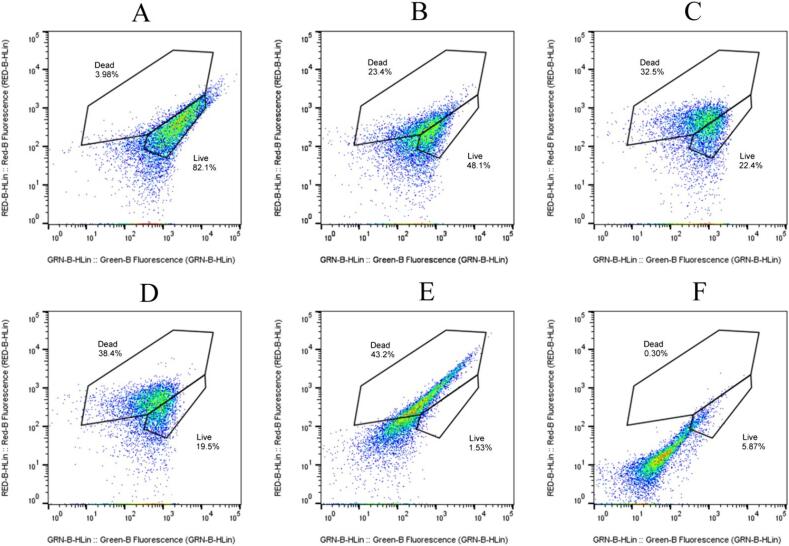
Flow cytometry analysis of viability changes in TS-treated *S. putrefaciens* using SYTO9 and PI fluorescent staining. The control group for normal growth without TS treatment (A), TS treatment: TS-70-100 (B), TS-70-150 (C), TS-70-200 (D), TS-70-250 (E), and TS-70-300 (F).

We employed flow cytometry and trypan blue staining to analyze the survival status of *S. putrefaciens* after TS treatment. Flow cytometry results (Fig. 2) showed that *S. putrefaciens* exhibited a significant trailing behavior with TS treatments (TS-70-100, TS-70-150, TS-70-200, TS-70-250, TS-70-300), indicating an increase in fragmented cellular material. This was consistent with our TEM, PCR results (Fig. 4, Fig. 5A) that showed cell wall disruption, DNA degradation, and leakage of intracellular substances following TS treatments. While we still found that the survival rate at TS-70-300 was higher than that at TS-70–250 (5.87 % vs. 1.53 %).

**Fig. 3:**
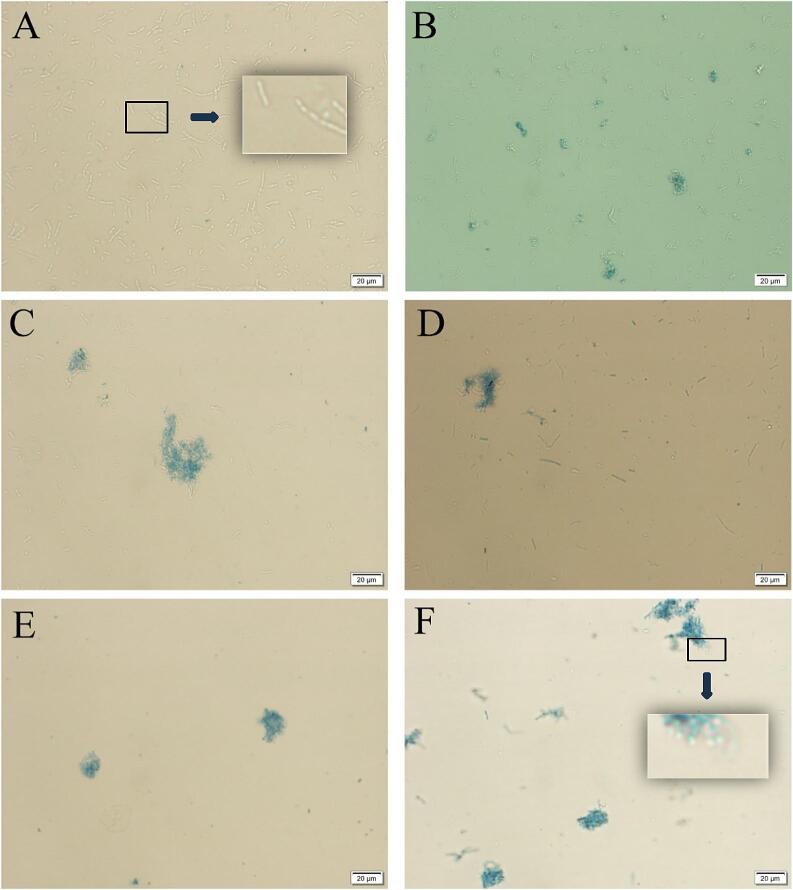
Trypan blue staining results after treating *S. putrefaciens* with different ultrasonic power. The control group for normal growth without TS treatment (A), TS-70-100 (B), TS-70-150 (C), TS-70-200 (D), TS-70-250 (E), and TS-70-300 (F).

**Fig. 4:**
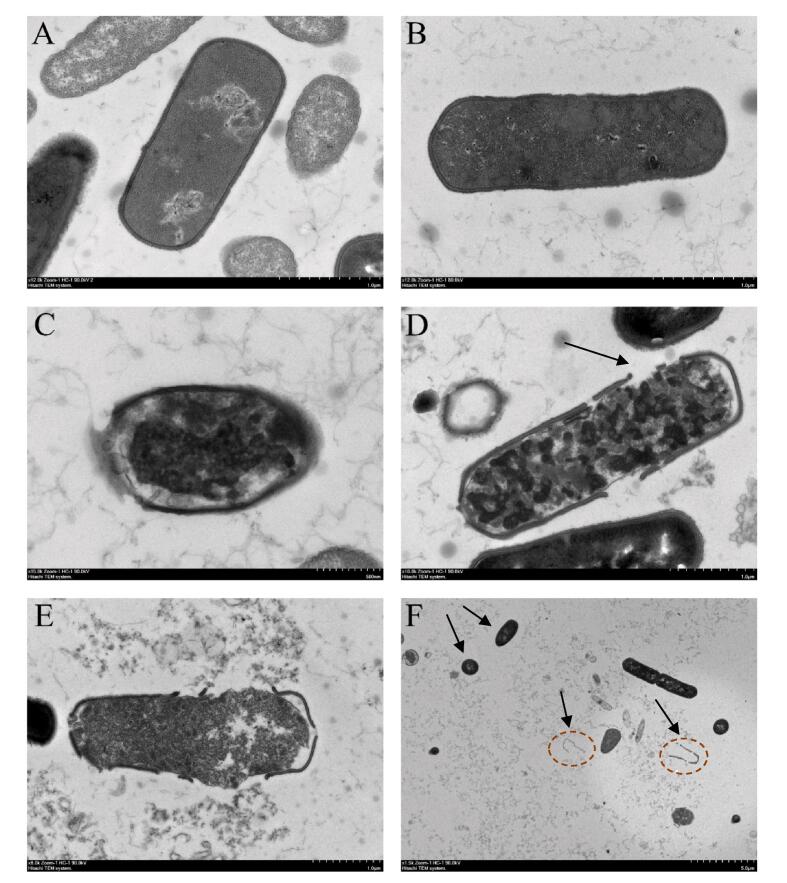
TEM images of *S. putrefaciens* after TS treatment. The control group for normal growth without TS treatment (A), TS-70-100 (B), TS-70-150 (C), TS-70-200 (D), TS-70-250 (E), and TS-70-300 (F).

We used trypan blue to strain the bacteria to further observe the living *S. putrefaciens* under TS treatments. The result (Fig. 3) revealed that untreated bacteria showed normal rod-shaped morphology and were transparent (unstained). However, as power increased, the number of stained cells increased, including clusters of dead cells. Interestingly, there were a small number of unstained cells in the TS-70-300 group (Fig. 3F), which was inconsistent with our TSA plate culture results (Fig. 1B). Additionally, our TEM results (Fig. 4) revealed that the morphology of some cells remained intact at TS-70–300 group, which was especially intriguing.

### 3.3 Performance of *S. putrefaciens* under TS treatment

#### 3.3.1 Morphological changes

In this experiment, changes in *S. putrefaciens* morphology induced by TS treatments were observed using TEM (Fig. 4). The structure of the untreated bacteria remained intact, with a tight cell wall, uniform cytoplasm, and a clear nuclear area. After TS treatments, significant changes in bacterial morphology were observed. At ultrasonic power of 100 (Fig. 4B) and 150 W (Fig. 4C), the morphology of bacteria was still relatively intact, indicating that *S. putrefaciens* was able to resist the pressure under these conditions. At power of 200 W (Fig. 4D) and 250 W (Fig. 4E), damage to the cell wall and cell membrane of *S. putrefaciens* was more severe, and intracellular substances began to leak. A large amount of intracellular substances leaked at 300 W (Fig. 4F), accompanied by the appearance of large cavities and many cell fragments, which is consistent with the results of Luo et al, who observed bacterial fragments in TEM images after exposure of *E. coli* to TS for 8 h [29]. This phenomenon arises from the combined effects of TS treatments, which encompass both the mechanical vibration effects of ultrasound and the thermal effects [30], [31]. Ultrasound generates high-frequency compression and expansion fluctuations, creating local high-pressure and low-pressure regions that rupture the microbial cell membrane. Consequently, heat treatment can more effectively inactivate the cell, hindering material exchange between the interior and exterior of the cell and ultimately leading to microbial death.

Interestingly, even at 300 W, we still found a few bacteria showed relatively intact morphology which was consistent with the results of flow cytometry (Fig. 4F). Other studies also found that *E. coli* maintained cellular integrity when induced into a VBNC state by chlorination and chloramination [32]. Keeping cellular integrity is one of the traits of bacteria entering the VBNC state in response to external stress [33]. However, for *S. putrefaciens*, the mechanism by which it regulates itself to maintain cellular integrity under TS treatment remains unclear.

#### 3.3.2 In vitro amplification

**Fig. 5:**
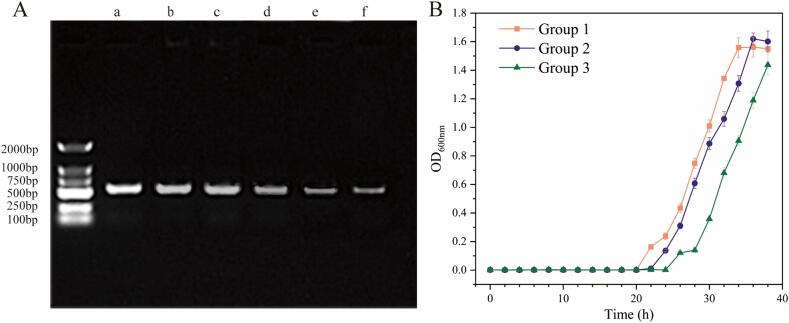
A:Changes in vitro amplification of *S. putrefaciens* DNA under TS treatment. The control group for normal growth without TS treatment (a), TS-70-100 (b), TS-70-150 (c), TS-70-200 (d), TS-70-250 (e), and TS-70-300 (f); B:Resuscitated of group TS-70-300.

Ultrasonic and heating are both leading to DNA break and assessment of its impact upon entering bacterial cells is crucial to advance research on the self-adaptive mechanism of response to ultrasonic and heating −induced stress. To explore the effect of TS treatments on the in vitro amplification of bacterial DNA, PCR reactions were performed using *S. putrefaciens* as the template. The electrophoresis bands in untreated samples were bright and clear, while the bands gradually faded with an increase in power (Fig. 5A). This occurred because the cavitation effect of ultrasound induced liquid cuts off the spiral chain, causing a decrease in DNA molecular weight [34].

Furthermore, we selected these bacteria (from group TS-70–300) for the resuscitation assay and found that they resuscitated and grew normally after 20–25 h (Fig. 5B). Fig. 2, Fig. 3F revealed that under TS-300–70 treatment *S. putrefaciens* still can survive, but from our TSA plate culture results (Fig. 1B), there was no visible colony growth. Thus, we determine those bacteria as VBNC state [35], [36]. While, the molecular mechanisms for bacteria resuscitation under DNA damage caused by ultrasound and heat treatment are still unknown.

### 3.4 4D-DIA Proteomic analysis of *S. putrefaciens* in the VBNC state

#### 3.4.1 Identification of differentially expressed proteins (DEPs)

Protein samples from strains in the VBNC state (case) and under normal culture conditions (control) were processed and analyzed, resulting in the identification of 5,396 peptide segments and 1,437 proteins. Venn diagram analysis of the protein overlap between the case and control groups revealed 1,418 proteins common to both groups (Figure S1). Comparison with the GO database revealed that the proteins were mainly involved in cellular processes, metabolic processes, biological regulation, localization, and stimuli responses (Figure S2).

**Fig. 6:**
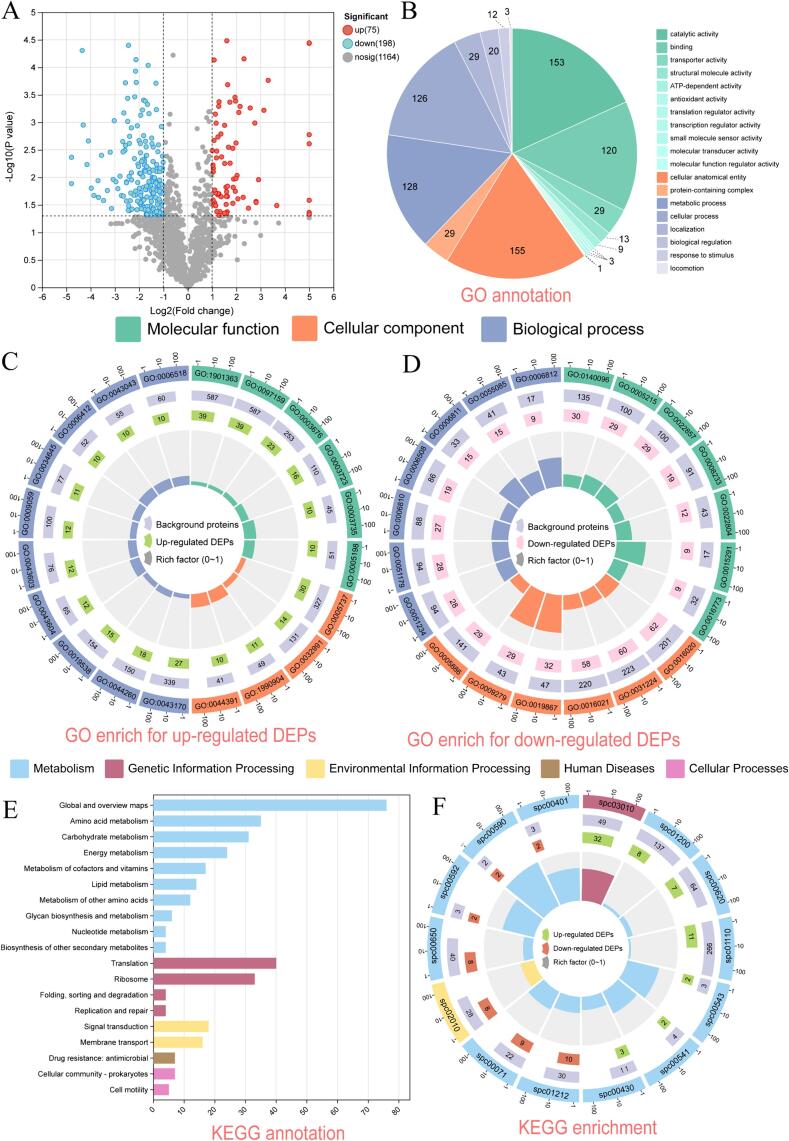
Analysis of differential proteins between the control and treatment groups. A: Volcano plot. B: GO annotation, displaying the distribution of DEPs in 19 GO terms. C–D: GO enrichment analysis of up-regulated DEPs and down-regulated DEPs, the outermost circle is the top 20 enriched GO terms, the second circle is the background proteins, the third circle is the count of up-regulated or down-regulated DEPs; the fourth circle exhibited the rich factor value for each GO term. E: KEGG annotation of DEPs F: KEGG enrichment analysis of DEPs, the outermost circle is all significantly enriched KEGG pathways, the second circle is the background proteins, the third circle is the count of up-regulated (green) or down-regulated DEPs (red), The fourth circle is enrichment factor.

Using *t*-tests, 75 upregulated and 198 downregulated proteins were identified (Table S2 and 3), including 8 unique proteins in the case group and 11 unique proteins in the control group (Fig. 6A). The cellular locations of the proteins were predicted based on the GO annotations (Fig. 6B) and subcellular localization data (Figure S3). The DEP functions included catalytic activity (153 DEPs), binding (120 DEPs), transporter activity (29 DEPs), structural molecule activity (13 DEPs), and ATP-dependent activity (13 DEPs) (Fig. 6B). Table S1 presents detailed information on the DEPs. The KEGG pathway annotation analysis revealed that the DEPs were assigned to 19 KEGG pathways, with a predominant association toward metabolism (Fig. 6E).

#### 3.4.2 Functional analysis of upregulated proteins

Fig. 6C shows the results of the GO enrichment analysis of the upregulated DEPs, which were mainly enriched in ribosomes, biosynthesis, stress response, and macromolecule metabolism. Among these, 32 annotations were related to ribosomal protein synthesis function, including rpsA, rplA, rpoA, rpsB, signal recognition particles (SRPs), and other ribosomal transcription regulatory protein families. Durack et al. also found a significant increase in ribosomal protein-related genes in *Listeria monocytogenes* after entering a sublethal state under conditions of high-osmotic or low-temperature stress [37]. Highly enriched expression of ribosomes implies an increased rate of protein synthesis [38], [39]. This may be because with TS treatments, *S. putrefaciens* needs to produce more stress proteins to respond to stimuli, maximize its survival, and subsequently enter the VBNC state. Reports have also indicated that ribosomal proteins act as independent peptides that are responsible for DNA repair and transcription [40].

KEGG enrichment analysis (Fig. 6F) revealed significant upregulation of the ribosomal and pyruvic acid pathways, which was consistent with the GO analysis results. Pyruvic acid is a crucial product of glycolysis [41], and changes in its metabolic pathway may indicate an acceleration of energy metabolism. Among the upregulated proteins, glutamine-fructose-6-phosphate aminotransferase (GlmS) is involved in glycolysis. GlmS participates in glutamine and amino sugar metabolism, those changes can produce more ATP [42]. It was reported that genes related to carbohydrate metabolism and transport, energy production, and peptidoglycan degradation were upregulated after *Pseudomonas syringae* pv*. syringae* entered the VBNC state induced by acetosyringone [43]. In this study, *S. putrefaciens* was treated with TS treatment, bacteria upregulated the glycolysis and produced more ATP to response the damage from ultrasonic and heating. This indicated that accelerating the generation of ATP is an adaptive strategy for bacteria to enter the VBNC state under harsh environmental conditions. In the future, inhibiting the production of ATP may be a method to control bacteria entering VBNC state, such as: ATP inhibitor [44].

The unique proteins in the case group included some repair-related functional proteins such as cytochrome *c*, deoxyribodipyrimidine photolyase, and ornithine decarboxylase SpeF (SpeF). Cytochrome *c* and deoxyribodipyrimidine photolyase maintain DNA structure, effectively identify DNA damage, and repair it [45], [46]. Considering that TS treatment causes *S. putrefaciens* DNA degradation and inhibits proliferation, it probably performs self-repair by overexpressing such proteins. Among these upregulated proteins, the expression abundance of SpeF was the highest, with a fold change of 9.929. SpeF catalyzes the decarboxylation of ornithine to produce putrescine, which is the first rate-limiting step in polyamine synthesis [47]. Polyamines bind to membrane proteins or phospholipids to maintain membrane stability [48]. Because TS treatment damages bacterial cell membranes, SpeF upregulation may be a response to ensure cellular integrity. SpeF is also involved in the antibiotic resistance mechanisms of various bacteria (DNA repair) [49]. For example, upregulation of SpeF expression in *E. coli* reduces the accumulation of intracellular reactive oxygen species caused by antibiotics, thereby preventing protein and DNA damage [50]. Therefore, the self-repair strategy is another adjustment made by *S. putrefaciens* to enter the VBNC state with TS treatments.

#### 3.4.3 Functional analysis of downregulated proteins

GO functional analysis of downregulated DEPs revealed that they were mainly enriched in membrane components, catalytic activity, and transport activity (Fig. 6D). Among the downregulated proteins, porin proteins (58 DEPs) were the most abundant, including those belonging to the OmpA, OmpW, and TolC families. OmpA is a nonspecific porin protein involved in the passive diffusion of various small molecules [51]. *Cronobacter sakazakii* strain Yrt2a upregulates OmpA under acid stress but downregulates it when the strain enters the unculturable state [52]. OmpW, a hydrophobic porin protein present in both the outer membrane and cytoplasm, is involved in various tolerance mechanisms, such as antibiotic resistance, osmotic stress, and low-oxygen stress [53]. Therefore, we speculate that *S. putrefaciens* decreases the expression of various membrane transport proteins to mitigate damage from TS treatments by reducing energy consumption [54], thereby entering the VBNC state. Additionally, TonB-dependent receptors (TBDRs) were significantly downregulated. TBDRs mainly assist in the transport of essential nutrients, which are either of sizeable molecular size or components of large compounds [55]. Thus, *S. putrefaciens* likely decreases its interaction with the external environment by reducing the expression of such proteins, subsequently lowering metabolic activity.

Interestingly, this study found that protein families involved in the resistance-nodulation-cell division (RND) efflux system, such as the TolC family and ABC transport proteins, were significantly downregulated. The RND efflux system is a mechanism of bacterial self-purification [56]. When a drug exerts an effect on a bacterium, the efflux system participates in the removal of toxic substances from inside the cell, protecting the bacterium from its own toxins [57]. For example, *E. coli* extrudes various antibacterial molecules with different chemical properties through the RND efflux system to reduce their toxicity [56]. The significant downregulation of the aforementioned transport proteins indicates that, to some extent, the inhibition of nutrient intake and efflux of harmful substances affects the normal metabolic activity of *S. putrefaciens*. This founding can provide some insights in future studies, such as: combine TS treatments with some natural antimicrobial substances which were influence by RND efflux system including: chitosan oligosaccharide, antimicrobial peptides etc.

KEGG pathway enrichment analysis (Fig. 6F) revealed significant changes in fatty acid degradation and metabolism pathways. The metabolism of fatty acids within cells generates phospholipids, which are crucial for cell-signaling pathways [58]. As part of energy metabolism, the downregulation of fatty acid metabolism (such as dehydrogenase) and degradation may indicate a decrease in overall metabolic levels. Laura et al. reported the induction of *Cupriavidus metallidurans* into a VBNC state with significantly decreased bacterial metabolic activity under conditions of water and nutrient deprivation stress [59].

Normally, the synthesis of fatty acid consume ATP and it is related to glycolysis, since glycolysis breaks down glucose to provide the acetyl-coa units required for fatty acid synthesis [60]. Our results revealed that glycolysis was upregulated and fatty acid was downregulated, this may because that: 1) fatty acid is a source of energy in the cells and storage units, *S. putrefaciens* needs to store more energy to cope with the complex environment, thus slowing the progress of the fatty acid metabolism; 2) fatty acid metabolism requires a large amount of oxygen [61], in this study, *S. putrefaciens* was exposed to ultrasound and heating, which contained little oxygen, and therefore, fatty acid metabolism was relatively low [62].

#### 3.4.4 PPI network analysis of DEPs

**Fig. 7:**
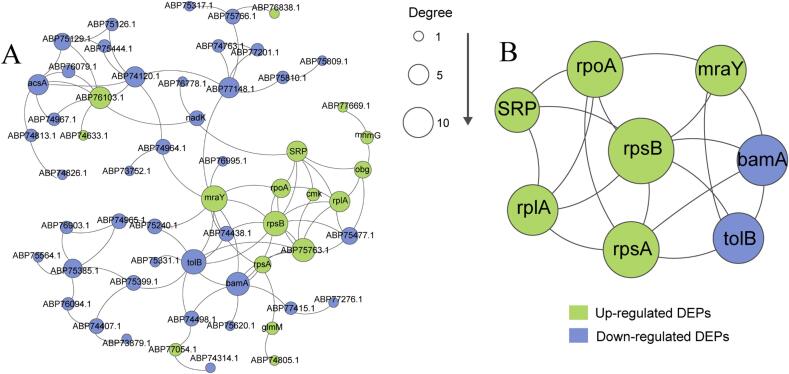
PPI network of all DEPs (A) and hub proteins (B) between control and treatment groups, each node represented a protein, and edges indicated protein interactions.

PPI network analysis identified the hub proteins for *S. putrefaciens* entering the VBNC state under TS treatment as rpsA, rplA, rpoA, rpsB, MraY, tolB, and SRPs (Fig. 7). Among these, rpsA, rplA, rpoA, rpsB, and SRPs belong to the ribosomal transcriptional regulatory protein family, which is consistent with our GO functional enrichment results. SRPs are ribonucleoprotein complexes that are responsible for delivering nascent peptide chains to membrane channels. Most bacterial membrane proteins are guided to their respective sites by SRPs [63]. We hypothesize that *S. putrefaciens* may produce a large number of stress proteins by upregulating the ribosomal protein family (particularly rpsB) and rely on SRPs to deliver them to the appropriate site, which are essential characteristics of bacteria entering the VBNC state [64], [65]. This supports our finding that the ribosomal transcriptional regulatory protein family serves as hub proteins.

Notably, the MraY protein is a key enzyme for cell wall synthesis, and abnormal MraY protein expression can result in the inability of the bacteria to synthesize cell walls, leading to cell lysis [66]. Previous TEM results have shown that TS treatments disrupt *S. putrefaciens* cell walls, consequently affecting the normal physiological activities of bacteria. Therefore, we speculate that this is also a strategy for *S. putrefaciens* to resist TS treatments damages and ensure its integrity.

In summary, with TS treatments, *S. putrefaciens* may primarily regulate its entry into the VBNC state via the following strategies: 1) upregulating the ribosomal protein family to guide the expression levels of relevant stress proteins and direct the localization of related proteins through SRPs to resist TS treatment; 2) upregulating related repair proteins, such as SpeF (polyamine secretion) and MraY protein (cell wall repair), to mitigate TS-induced damage; 3) upregulating catalytic enzymes, such as GlmS, to accelerate glycolysis, thereby producing ATP; and 4) downregulating transport proteins and energy metabolism functional proteins to enter into the VBNC state. Our findings reveal the complex response mechanism of bacteria to TS treatments interference and provide strategies for subsequent efforts to reduce the development of sublethal-state putrefactive bacteria in aquatic product sterilization and achieve efficient sterilization.

## 4 Conclusion

In this study, we verified the VBNC state of *S. putrefaciens* after TS treatments and analyzed its regulation mechanisms. Our results found that *S. putrefaciens* still enter the VBNC state after TS treatment (300 W, 70 °C, 30 min). Moreover, VBNC state of *S. putrefaciens* can resist the damage caused by TS treatments, such as: cell wall break, DNA degradation, etc. Using 4D-DIA proteomics technology, we discovered that *S. putrefaciens* responded to TS teatment by upregulating stress protein expression, initiating the repair of TS-induced damage by relevant proteins, accelerating ATP producing through glycolysis, and downregulating translocators and energy metabolism proteins, ultimately leading to entry into the VBNC state. Additionally, PPI network analysis revealed the central role inducing *S. putrefaciens* entering VBNC status may be the rpsB which regulates the translation level of functional proteins. Our findings provide a new perspective on the resistance mechanisms of spoilage bacteria to TS treatments, offering new strategies for aquatic product sterilization and bacterial resistance management based on molecular mechanisms. These will lead to an improvement in food safety and extend the shelf life of aquatic products in TS treatments. In the future, interventions like chemical inhibitors could be used to block the expression of proteins such as rpsB, SRP, MraY, and SpeF. This would prevent bacteria from entering the VBNC state, allowing for the complete eradication of S. putrefaciens.

## CRediT authorship contribution statement

**Ziwei Jiang:** Writing – original draft, Formal analysis, Data curation. **Yi Wang:** Validation, Methodology. **Shunjie Bai:** Investigation. **Chan Bai:** Validation, Methodology. **Ziyi Tu:** Funding acquisition. **Hailan Li:** Validation, Methodology. **Peng Guo:** Funding acquisition. **Tao Liao:** Funding acquisition. **Liang Qiu:** Writing – review & editing, Supervision, Funding acquisition.

## Declaration of competing interest

The authors declare that they have no known competing financial interests or personal relationships that could have appeared to influence the work reported in this paper.
