# Supplementary material for: The viable but non-culturable (VBNC) status of Shewanella putrefaciens (S. putrefaciens) with thermosonication (TS) treatment
Source: Ultrason Sonochem. 2024 Jul 30;109:107008. doi: 10.1016/j.ultsonch.2024.107008 (PMC11345692; doi:10.1016/j.ultsonch.2024.107008)
Supplement: Supplementary Data 1 [file mmc1.docx]

Table S1 DEPs of *S. putrefaciens* entering VBNC state under TS treatment (part)

| No. | Accession number | Protein | Fold change(case/control) | p-value(case/control) | Domain Description | Subcellular location |
| --- | --- | --- | --- | --- | --- | --- |
| Response to stress | | | | | | |
| 1 | QXN24143.1; QYX63866.1 | molecular chaperone DnaJ | 5.938↑; 2.622↑ | 0.0005386; 0.004248 | DnaJ C terminal domain; | CYT |
| 2 | GGN24257.1 | deoxyribodipyrimidine photo-lyase | 32↑ | 0.00002657 | FAD binding domain of DNA photolyase | CYT |
| 3 | GGN21653.1 | peroxiredoxin | 7.019↑ | 0.02739 | AhpC/TSA family | CYT |
| 4 | QXN24189.1 | catalase KatB | 4.87↑ | 0.02304 | Catalase; | PLA |
| 5 | QXN25583.1 | catalase/peroxidase HPI | 2.863↑ | 0.001125 | Peroxidase | CYT |
| 6 | GGN14894.1 | endopeptidase La | 2.161↑ | 0.02912 | Lon protease | CYT |
| 7 | GGN12015.1 | glutamine--fructose-6-phosphate aminotransferase | 32↑ | 0.001666 | SIS domain | CYT |
| 8 | AVV85152.1 | cytochrome C | 32↑ | 0.04337 | Cytochrome_C7 | CYT |
| 9 | QXN25298.1 | ornithine decarboxylase SpeF | 9.929↑ | 0.0001624 | Orn/Lys/Arg decarboxylase, major domain; | CYT |
| Protein folding |  |  |  |  |  |  |
| 7 | QYX64304.1 | FKBP-type peptidyl-prolyl cis-trans isomerase | 8.783↑ | 0.0005942 | Domain amino terminal to FKBP-type peptidyl-prolyl isomerase | CYT |
| Cell wall repair | | | | | | |
| 8 | QYX63035.1 | endolytic transglycosylaseMltG | 2.854↑ | 0.04481 | YceG-like family | CYT |
| 9 | GGN28306.1 | phospho-N-acetylmuramoyl-pentapeptide-transferase | 3.2261↑ | 0.02102 | Glycosyl transferase family | CYT |
| Ribosome | | | | | | |
| 9 | QXN25236.1 | 50S ribosomal protein L24 | 4.3↑ | 0.0006514 | Ribosomal proteins 50S L24/mitochondrial 39S L24 | CYT |
| 10 | QXN24386.1 | 50S ribosomal protein L13 | 3.88↑ | 0.01786 | Ribosomal protein L13 | CYT |
| 11 | QXN25226.1 | 50S ribosomal protein L3 | 3.702↑ | 0.01073 | Ribosomal protein L3 | CYT |
| 12 | GGN22295.1 | 50S ribosomal protein L27 | 3.664↑ | 0.01518 | Ribosomal L27 protein | CYT |
| 13 | WP_198781920.1 | 50S ribosomal protein L1 | 3.099↑ | 0.02224 | Ribosomal protein L1p/L10e family | CYT |
| 14 | GGN13915.1 | 30S ribosomal protein S2 | 2.367↑ | 0.02083 | Ribosomal protein S2 | CYT |
| 15 | QYX73687.1 | Obg family GTPase CgtA | 2.363↑ | 0.00184 | 50S ribosome-binding GTPase | CYT |
| 16 | A4YBX8.1 | RL22_SHEPC RecName: Full=Large ribosomal subunit protein uL22; | 2.287↑ | 0.003323 | Ribosomal protein L22p/L17e | CYT |
| Export from cell | | | | | | |
| 17 | GGN07012.1 | Type IV pilus secretin PilQ | 0.4102↓ | 0.01164 | Secretin | CYT |
| 18 | QXN25185.1;GGN31581.1 | type II secretion system secretin GspD | 0↓; 0.3529↓ | 0.002617;0.01769 | Bacterial type II and III secretion system protein | CYT |
| Drug resistance | | | | | | |
| 19 | GGN28306.1 | phospho-N-acetylmuramoyl-pentapeptide-transferase | 0.2261↓ | 0.02102 | Glycosyl transferase family 4 | CYT |
| 20 | AVV84641.1 | multidrug transporter | 0.3108↓ | 0.03683 | AcrB/AcrD/AcrF family | CYT |
| 21 | QXN24668.1 | TolC family outer membrane protein | 0.2595↓ | 0.003576 | Outer membrane efflux protein | CYT |
| 22 | QXN23310.1 | phosphoethanolamine--lipid A transferase | 0.4265↓ | 0.003401 | Phosphoethanolamine transferase EptA/EptB | CYT |
| 23 | GGN09384.1 | N-acetylmuramoyl-L-alanine amidase | 0.4334↓ | 0.002556 | N-acetylmuramoyl-L-alanine amidase | CYT |
| 24 | GGN20379.1;QXN24668.1 | outer membrane channel protein TolC | 0.2412↓;0.2595↓ | 0.005737;0.003576 | Outer membrane efflux protein | CYT |
| Outer membrane | | | | | | |
| 25 | GGN11825.1 | outer membrane protein OmpA | 0.151↓ | 0.0214 | OmpA family | CYT |
| 26 | GGN20000.1 | OmpA family lipoprotein | 0.2908↓ | 0.00588 | OmpA family | CYT |
| 27 | QYX66159.1 | outer membrane protein OmpW | 0.04916↓ | 0.00003954 | OmpW family | CYT |
| 28 | QYX63194.1;QXN26042.1;WP_011789640.1 | TonB-dependent receptor | 0↓;0↓;0.0361↓ | 0.01422;0.00166; 0.01299 | TonB-dependent Receptor Plug Domain | CYT |
| 29 | GGN13981.1 | outer membrane protein assembly factor BamA | 0.4427↓ | 0.04081 | Omp85 superfamily domain | CYT |
| ABC transporters | | | | | | |
| 30 | GGN15431.1 | lipid A export ATP-binding/permease protein MsbA | 0.2402↓ | 0.01244 | ABC transporter transmembrane region | CYT |
| 31 | QYX65329.1 | excinuclease ABC subunit UvrA | 0↓ | 0.000003371 | UvrA interaction domain | CYT |
| 32 | WP_220592353.1 | LPS export ABC transporter permease LptG | 0.2622↓ | 0.01905 | Lipopolysaccharide export system permease LptF/LptG | CYT |
| 33 | QXN25608.1 | ABC transporter ATP-binding protein/permease | 0.2593↓ | 0.002429 | ABC transporter transmembrane region | CYT |
| 34 | QXN25878.1 | ABC transporter ATP-binding protein/permease | 0.1886↓ | 0.002406 | ABC transporter transmembrane region | CYT |
| 35 | QXN24399.1 | lipid asymmetry maintenance ABC transporter permease subunit MlaE | 0.3948↓ | 0.04304 | Permease MlaE | CYT |


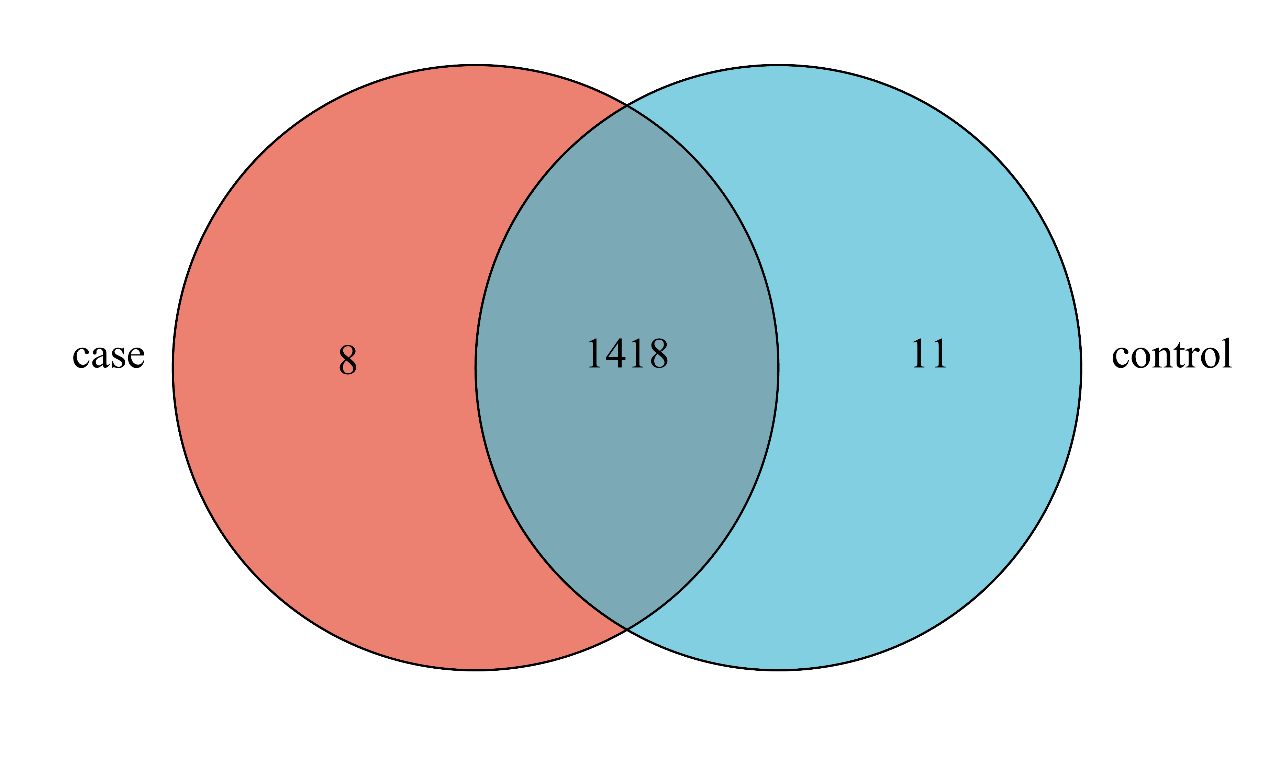


Figure S1 Venn diagram analysis of the protein overlap between the case and control groups


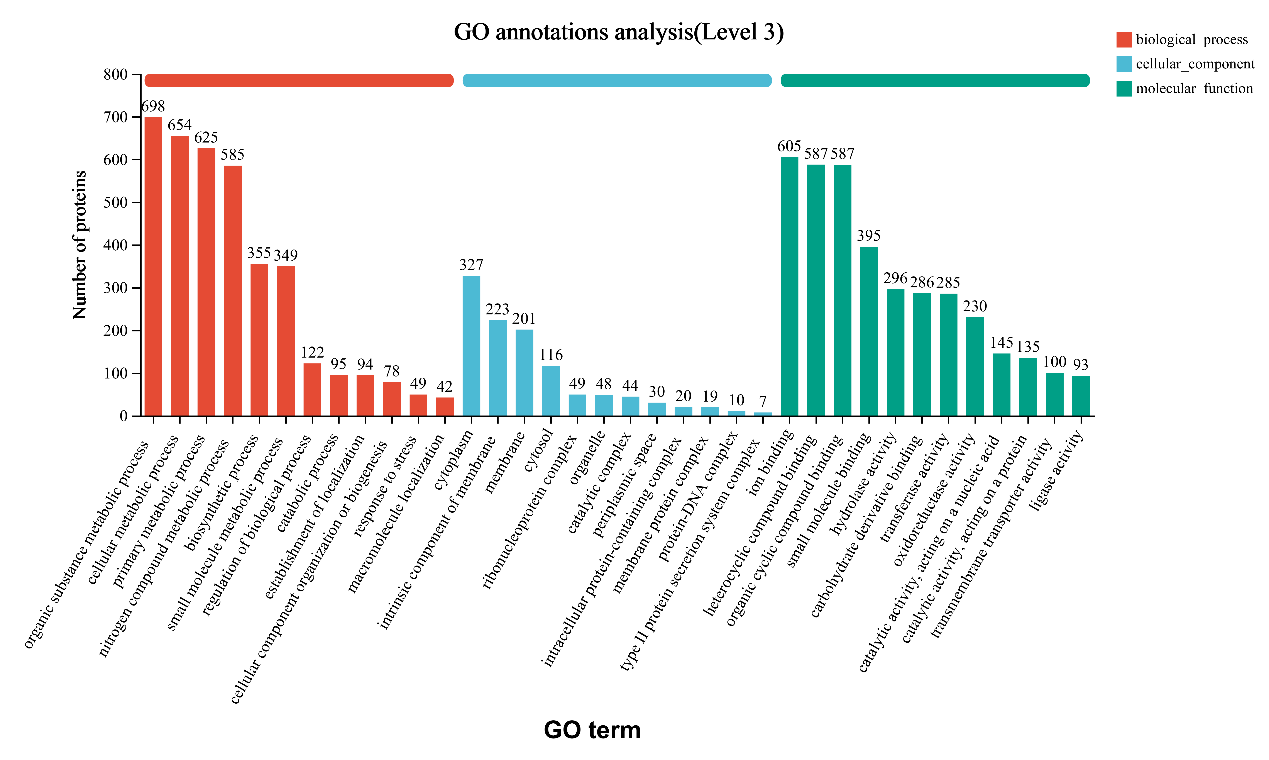


Figure S2 Go functional annotation of all proteins identified by proteomics


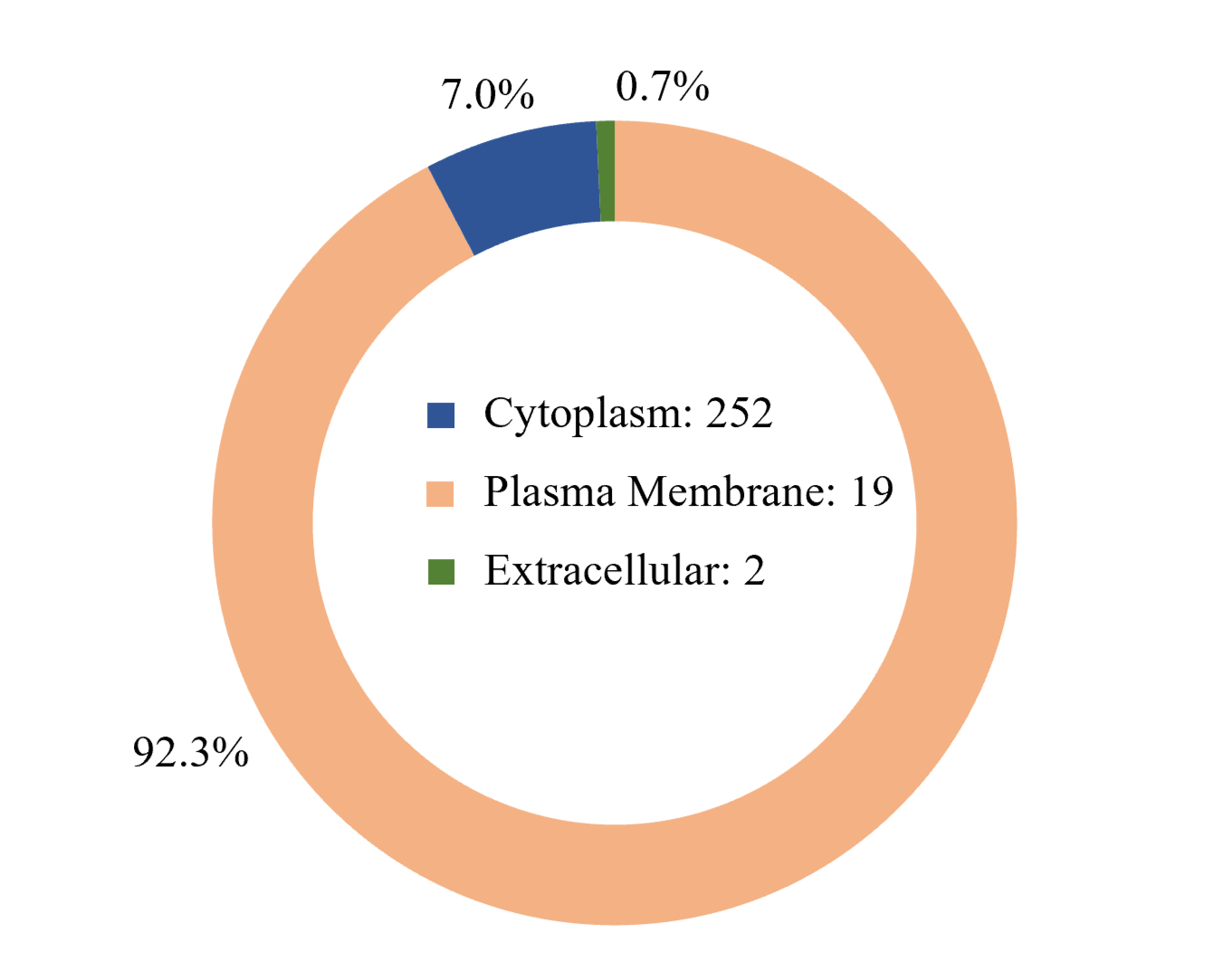


Figure S3 Subcellular localization data of DEPs

Table S2. Information on the top 20 significantly (corrected p-value < 0.05) enriched GO terms for up-regulated and down-regulated differentially expressed proteins (DEPs), respectively.

| **GO Term** | **Description** | **Number** **of DEPs** | **Regulation** |  |
| --- | --- | --- | --- | --- |
| **Molecular function** | | | |  |
| GO:1901363 | heterocyclic compound binding | 39 | Up |  |
| GO:0097159 | organic cyclic compound | 39 | Up |  |
| GO:0003676 | nucleic acid binding | 23 | Up |  |
| GO:0003723 | RNA binding | 16 | Up |  |
| GO:0003735 | structural constituent of ribosome | 10 | Up |  |
| GO:0005198 | structural molecule activity | 10 | Up |  |
| **Biological process** | | | |  |
| GO:0043170 | macromolecule metabolic process | 27 | Up |  |
| GO:0044260 | cellular macromolecule metabolic process | 18 | Up |  |
| GO:0019538 | protein metabolic process | 15 | Up |  |
| GO:0043604 | amide biosynthetic process | 12 | Up |  |
| GO:0043603 | cellular amide metabolic process | 12 | Up |  |
| GO:0009059 | macromolecule biosynthetic process | 12 | Up |  |
| GO:0034645 | cellular macromolecule biosynthetic process | 11 | Up |  |
| GO:0006412 | translation | 10 | Up |  |
| GO:0043043 | peptide biosynthetic process | 10 | Up |  |
| GO:0006518 | peptide metabolic process | 10 | Up |  |
| **Cellular component** | | | |  |
| GO:0005737 | cytoplasm | 30 | Up |  |
| GO:0032991 | protein-containing complex | 14 | Up |  |
| GO:1990904 | ribonucleoprotein complex | 11 | Up |  |
| GO:0044391 | ribosomal subunit | 10 | Up |  |
| **Molecular function** | | | |  |
| GO:0140096 | catalytic activity, acting on a protein | 30 | Down |  |
| GO:0005215 | transporter activity | 29 | Down |  |
| GO:0022857 | transmembrane transporter activity | 29 | Down |  |
| GO:0008233 | peptidase activity | 19 | Down |  |
| GO:0022804 | active transmembrane transporter activity | 12 | Down |  |
| GO:0015291 | secondary active transmembrane transporter activity | 9 | Down |  |
| GO:0016773 | phosphotransferase activity, alcohol group as acceptor | 9 | Down |  |
| **Biological process** | | | | |
| GO:0051234 | establishment of localization | 28 | Down |  |
| GO:0051179 | localization | 28 | Down |  |
| GO:0006810 | transport | 27 | Down |  |
| GO:0006508 | proteolysis | 19 | Down |  |
| GO:0055085 | transmembrane transport | 15 | Down |  |
| GO:0006812 | cation transport | 9 | Down |  |
| **Cellular component** | | | |  |
| GO:0016020 | membrane | 62 | Down |  |
| GO:0031224 | intrinsic component of membrane | 60 | Down |  |
| GO:0016021 | integral component of membrane | 58 | Down |  |
| GO:0019867 | outer membrane | 32 | Down |  |
| GO:0009279 | cell outer membrane | 29 | Down |  |
| GO:0005886 | plasma membrane | 29 | Down |  |
|  |  |  |  |  |

Table S3. Information on all significantly (corrected p-value < 0.05) enriched KEGG pathways for up-regulated and down-regulated differentially expressed proteins (DEPs), respectively.

| **KEGG Pathway** | **Description** | **Down-regulated DEPs** | **Up-regulated DEPs** |
| --- | --- | --- | --- |
| spc03010 | Ribosome | 0 | 32 |
| spc01200 | Carbon metabolism | 0 | 8 |
| spc00620 | Pyruvate metabolism | 0 | 7 |
| spc01110 | Biosynthesis of secondary metabolites | 0 | 11 |
| spc00543 | Exopolysaccharide biosynthesis | 0 | 2 |
| spc00541 | O-Antigen nucleotide sugar biosynthesis | 0 | 2 |
| spc00430 | Taurine and hypotaurine metabolism | 0 | 3 |
| spc01212 | Fatty acid metabolism | 10 | 0 |
| spc00071 | Fatty acid degradation | 9 | 0 |
| spc02010 | ABC transporters | 8 | 0 |
| spc00650 | Butanoate metabolism | 8 | 0 |
| spc00592 | alpha-Linolenic acid metabolism | 2 | 0 |
| spc00590 | Arachidonic acid metabolism | 2 | 0 |
| spc00401 | Novobiocin biosynthesis | 2 | 0 |
